# Acute Pancreatitis as an Extrahepatic Complication of Acute Hepatitis E Infection: A Case Report

**DOI:** 10.1002/ccr3.72658

**Published:** 2026-05-04

**Authors:** Syed Abdur Rehman Shah, Intikhab Alam, Syed Noman Ali Shah, Umaima Cheema, Aymar Akilimali

**Affiliations:** ^1^ Department of Medicine Khyber Medical College/Affiliated Teaching Hospital Peshawar Pakistan; ^2^ King Edward Medical University Lahore Pakistan; ^3^ Medical Research Circle Goma Congo

**Keywords:** acute pancreatitis, acute viral hepatitis, case report, extrahepatic manifestation, hepatitis E virus

## Abstract

Clinicians should consider Hepatitis E Virus (HEV) a rare but significant etiology alongside established primary triggers, including gallstones and alcohol. Early HEV screening for hepatic and pancreatic dysfunction is required to improve care and avoid unnecessary interventions, especially in endemic regions.

## 1 Introduction

Acute pancreatitis is a common and potentially serious condition characterized by abrupt inflammation of the pancreas. Although the diagnosis of acute pancreatitis typically focuses on characteristic symptoms and elevated pancreatic enzymes, predicting clinical progression and long‐term outcomes remains difficult. Early identification of high‐risk patients is critical to determine various aspects, like the appropriate level of care required for the respective patient, the need for intensive monitoring, and the timing of targeted interventions. Gallstones and alcohol use account for approximately 35% to 40% and 17% to 25% of acute pancreatitis cases in the United States (US), respectively [1, 2]. While gallstones and alcoholism are widely known to be the most common causative agents of acute pancreatitis, about 10% of cases are thought to be caused by infectious microorganisms. These microorganisms include viruses (e.g., mumps, Coxsackie B, and hepatitis), bacteria (e.g., *Mycoplasma pneumoniae* and leptospirosis), and parasites (e.g., Ascaris lumbricoides, *Fasciola hepatica* , and hydatid disease) [3]. In 1944, LINSEY was the first researcher to report the association between acute pancreatitis and infectious hepatitis. This well‐known association is more frequently seen in settings where hepatitis is due to serotype A (HAV); needless to say, other serotypes, like hepatitis B (HBV) and C (HCV), have also been reported with quite a few infections as well [4, 5, 6, 7, 8, 9].

We present a case of a 35‐year‐old Pashtun Male with no previous comorbidities who presented with epigastric pain, jaundice, and vomiting. He was eventually diagnosed with acute hepatitis, along with acute pancreatitis. The etiology was unique and challenging to determine, as all of his labs and imaging negated the common causes leading to pancreatitis.

## 2 Case Presentation

### 2.1 Case History

**FIGURE 1:**
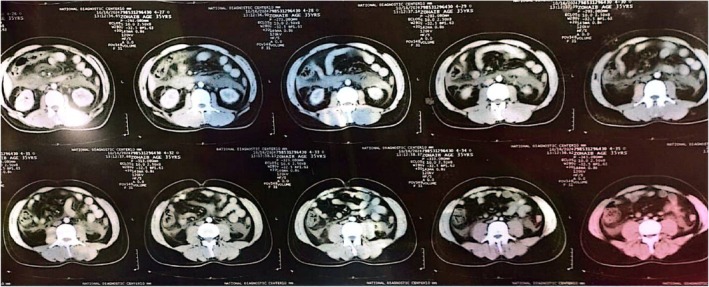
Axial Abdominal CT Scan (Upper Section) Showing an edematous pancreas with significant peripancreatic fluid collections and fat stranding.

**FIGURE 2:**
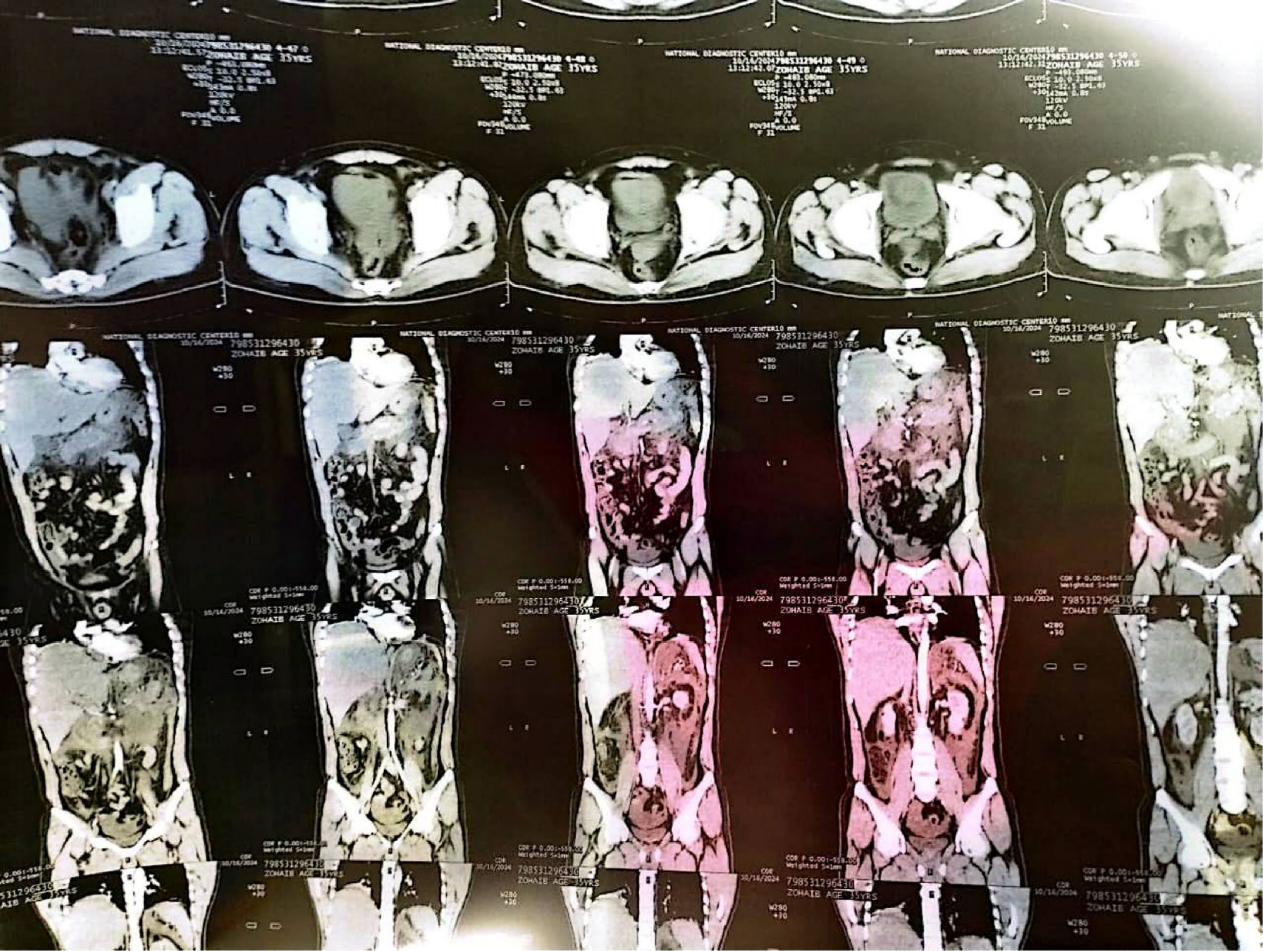
Axial Abdominal CT Scan (Mid Section) Revealing persistent pancreatic inflammation and the beginning of fluid extension into the paracolic gutters.

**FIGURE 3:**
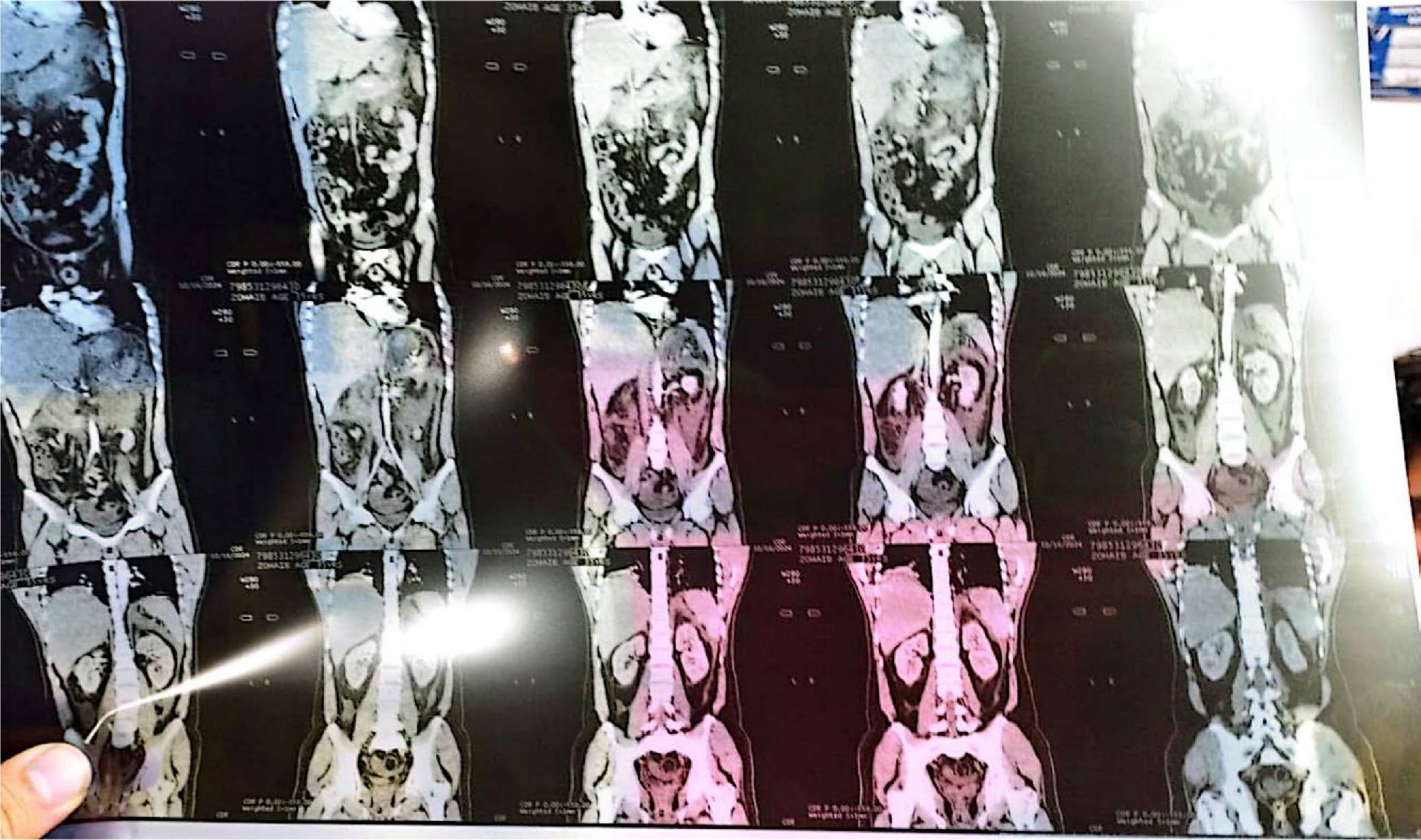
Coronal Reformatted CT Scan Demonstrating the longitudinal extent of pancreatic swelling and the presence of mild to moderate abdomino‐pelvic ascites.

A 35‐year‐old adult male with chief complaints of nausea and jaundice for 3 days duration, with a recent onset of mild abdominal pain and vomiting, was admitted to the ward. There was no history of alcohol use, gallstone disease, causative drugs, or trauma. The patient denies a history of any blood transfusion, sexual intercourse, or addictive drugs. At the time of presentation, his pulse was regular (98 beats per minute), blood pressure was 100/80 mmHg, and respiratory rate was 20/min. The patient was afebrile (Figures 1, 2, 3).

### 2.2 Examination

On general examination, the patient looked severely jaundiced and was in discomfort. He had scleral icterus, and her skin looked jaundiced. There was no cyanosis or edema. His abdominal examination upon inspection was normal, with reduced abdominal movements during respiration. Upon palpation, mild tenderness in the epigastric region with no guarding was seen. There were no palpable masses with no hepatomegaly or splenomegaly. Bowel sounds were diminished upon auscultation. Other systemic examination was unremarkable with normal heart sound on auscultation, and the chest was clear bilaterally. CNS examination was also normal; there was no hepatic flap or signs of hepatic encephalopathy or cerebral edema, and the patient was oriented in time, person, and place.

### 2.3 Investigation

**FIGURE 4:**
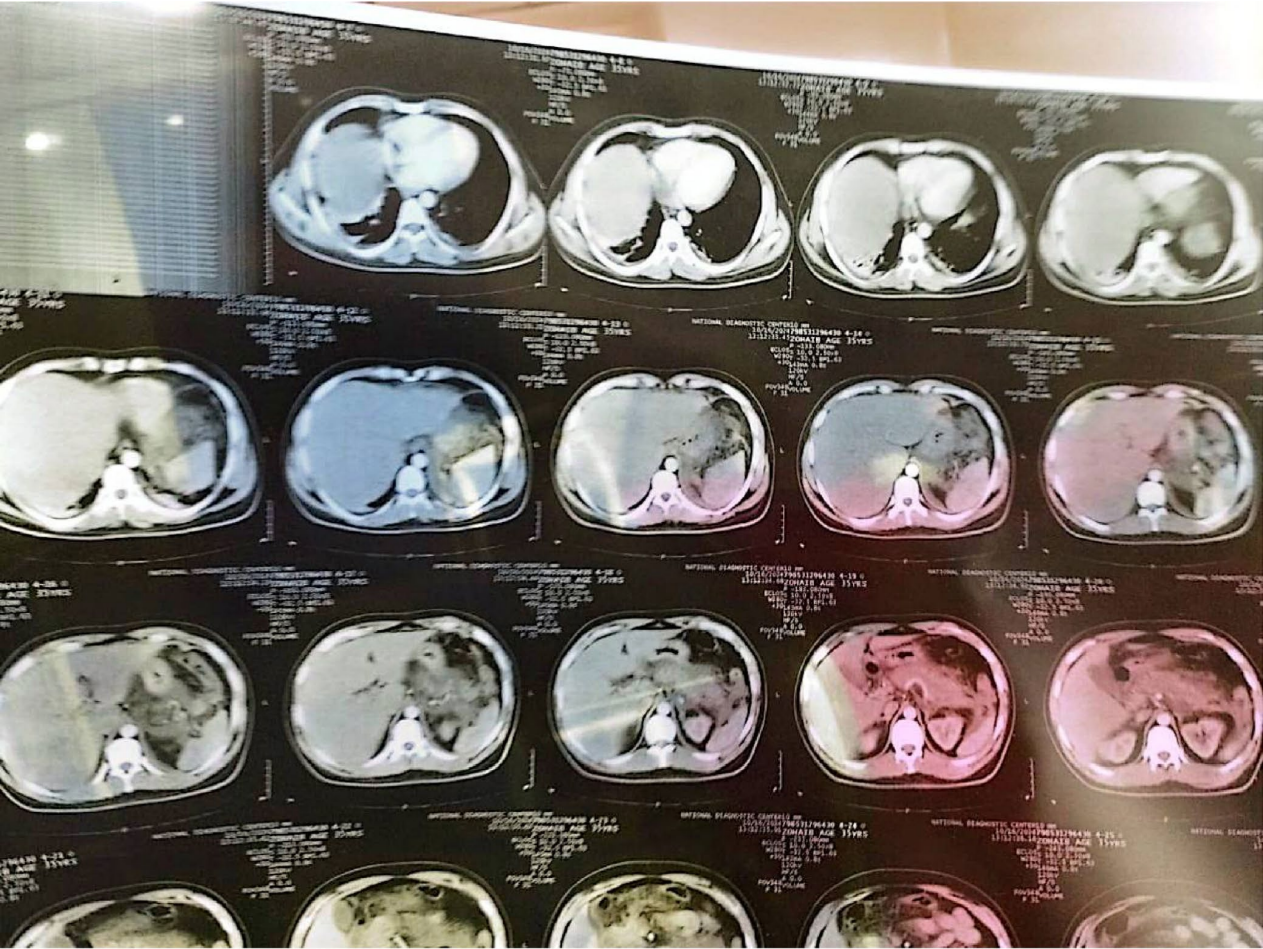
Upper Abdominal CT Sections Showing hepatomegaly (enlarged liver) and gallbladder wall thickening in the absence of gallstones (cholelithiasis).

**FIGURE 5:**
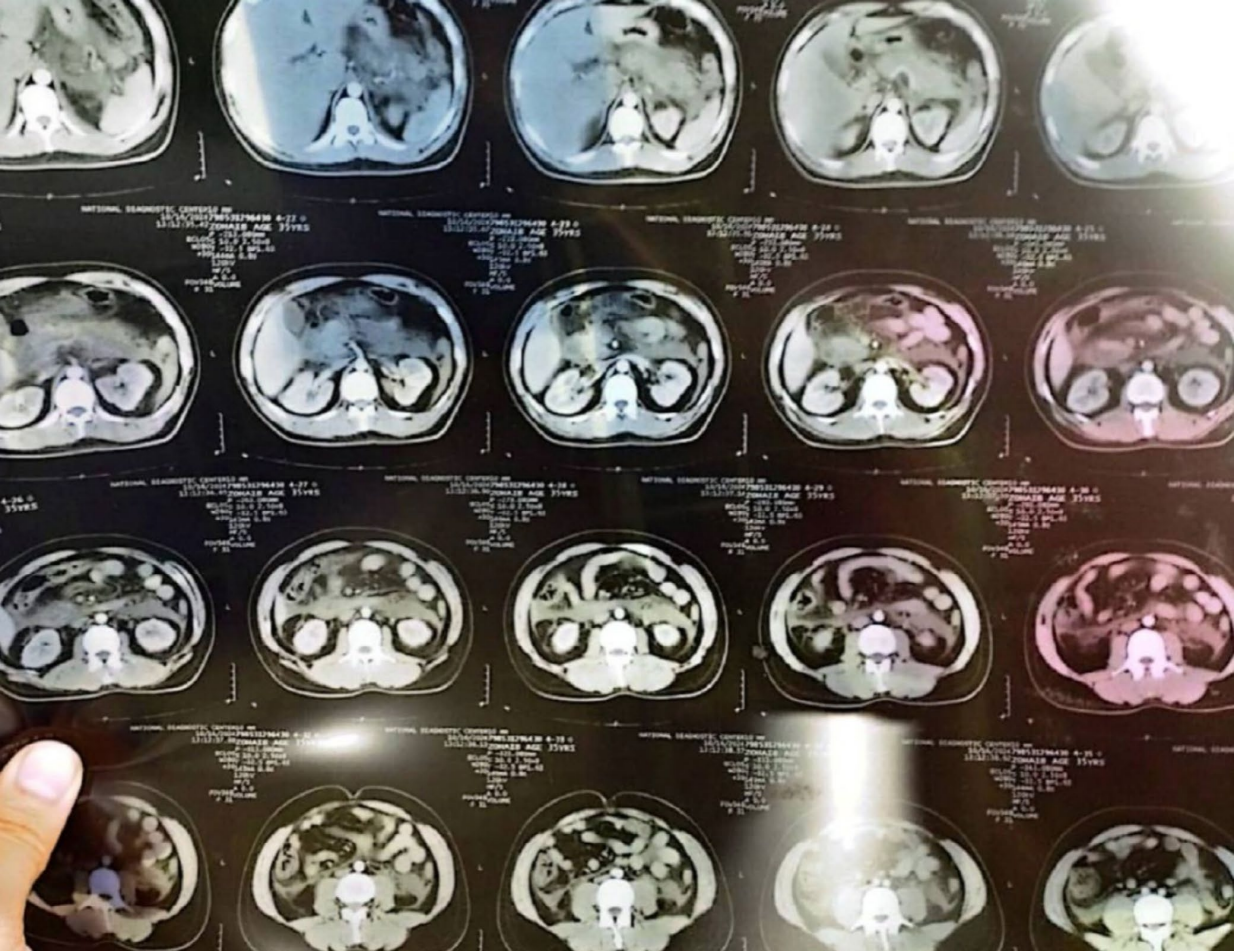
Pelvic CT Sections Confirming the absence of obstructive masses and evaluating the extent of free fluid within the pelvic cavity.

**FIGURE 6:**
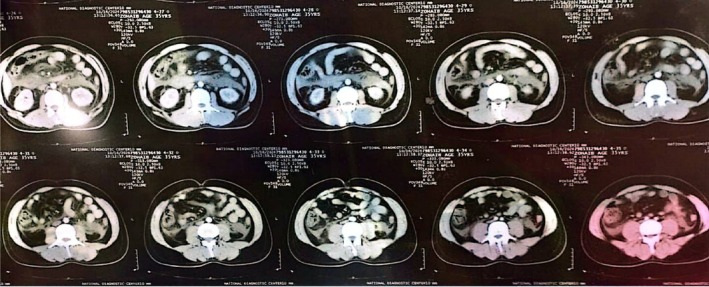
CT Scout/Overview Indicating patient positioning and preliminary localization of the hepatic and pancreatic distress areas.

Laboratory reports on arrival revealed abnormal liver function tests, which include serum bilirubin 25 mg/dL, SGPT of 1096 U/L, and serum alkaline phosphatase of 146 U/L. Other reports were normal, including CBC, RFTs, albumin, coagulation, and viral profile. Chest X‐ray and ECG did not reveal any abnormality. Emergency ultrasound examination of the abdomen revealed hepatomegaly with gallbladder wall thickening, along with no evidence of any cholelithiasis. The patient was admitted to the hospital under suspicion of acute hepatitis. On the 2nd day of admission, the patient developed severe abdominal pain and vomiting. His labs were done, which showed Elevated Amylase 242 and Lipase 407.7, PT 67 s, aPTT 54 s AND INR was 5.6. The patient was diagnosed with Acute Pancreatitis and Acute Liver Failure and was shifted to the ICU for critical care. There he stayed for 4 days, and on the 4th day his labs were as follows: Serum Bilirubin as 16, ALT as 61, and AlkPO4 as 101. Coagulation profile also came down to normal with PT 15 s, aPTT 30s, and INR 1.2. His amylase remained elevated at 244. He was shifted back to the ward and was started on treatment for Acute Pancreatitis. An extensive workup was carried out, and all relevant investigations were performed to explore the causative factors for pancreatitis. The ultrasound showed no gallstones. There was no history of alcohol use or illicit drug use. Normal Lipid Profile and his blood work for Hepatitis A, B, and C were negative. Serum IgM for Hep E came out positive. Antibody testing for autoimmune hepatitis was negative. Ceruloplasmin and copper levels were normal. Slit lamp examination did not reveal any Kayser‐Fleischer (K‐F) ring (Figures 4, 5, 6).

### 2.4 Differential Diagnosis

Other causes of infectious hepatitis, such as hepatitis A and B, were ruled out. Arboviral infections capable of causing hepatitis, such as malaria and dengue, were excluded. Other infections ruled out were varicella zoster, Epstein– Barr virus, CMV, echovirus, and Salmonella. Autoimmune causes of hepatitis were ruled out by respective antibody testing. Wilson's disease was unlikely in the setting of absent K‐F ring and normal ceruloplasmin levels. There was no history of substance abuse or alcohol addiction, which may have led to either hepatitis or pancreatitis. Hypertriglyceridemia, as a cause of pancreatitis, was excluded by a normal lipid profile. Connective tissue disorders were ruled out by relevant antibody testing (antinuclear antibody and antiphospholipid antibody).

### 2.5 Outcome and Follow‐Up

Serial monitoring of liver function tests, complete blood counts, amylase, lipase, and BUN showed improvement in parameters. An ultrasonogram of the abdomen repeated on day 10 revealed peri‐pancreatic oedema. A repeat chest X‐ray also showed spontaneous resolution of the pleural effusion. The patient was discharged following the clinical resolution of abdominal pain and jaundice, 2 weeks following admission. A month later, LFTs were within the normal limits, with the patient having an uneventful recovery accompanied by mild generalized weakness and a subnormal appetite.

## 3 Discussion

Acute pancreatitis has a wide range of severity and etiology. Gallstones and alcohol consumption account for the majority of cases in Western populations [1, 2]. Infectious aetiologies, although uncommon, comprise nearly 10% of cases and include viral, bacterial, and parasitic pathogens [3]. Among viral causes, hepatitis A virus (HAV) tends to be frequently reported, whereas hepatitis B (HBV) and C (HCV) are rarely seen [4, 5, 6, 7, 8]. Acute pancreatitis secondary to hepatitis E virus (HEV) infection is extremely rare, with very few cases documented in the literature [5, 9]. Our case serves to be a unique one as it observed severe symptoms requiring prompt and detailed investigation and follow‐up, compared to other cases present in the literature reporting relatively mild symptoms [5, 9].

In this case, we present a 35‐year‐old adult male with no previous comorbidities who developed concurrent acute hepatitis E and acute pancreatitis. The patient presented with jaundice, nausea, and vomiting. His pathology reports at that time revealed markedly elevated liver enzymes (ALT 1096 U/L, bilirubin 25 mg/dL), and we admitted him under a provisional diagnosis of Acute Hepatitis. Two days later, he developed severe epigastric pain, and his labs showed increased pancreatic enzymes (amylase 242 U/L, lipase 407.7 U/L). We excluded common causes such as gallstones, alcohol use, hypertriglyceridemia, drugs, and autoimmune hepatitis after extensive evaluation. Ultrasonography showed no gallstones, history and urine screen negated any drug abuse, lipid profile ruled out the possibility of hypertriglyceridemia, and labs observed no significant finding pointing toward autoimmune hepatitis, thus ruling out all other differentials, making our diagnosis most likely. His viral serology showed positive Hepatitis E virus antibody IgM.

Mishra et al. first described HEV‐associated pancreatitis as a typically self‐limiting disease, whereas in our case, a significant level of hepatic dysfunctions was observed, pointing towards acute liver failure [7]. Thus, our case is rare and important as it guides clinicians and healthcare professionals to remain vigilant in diagnosing pancreatic complications, even in cases like those presenting with severe HEV. Generally, such cases remain undertreated or misdiagnosed, especially in patients experiencing high‐grade coagulopathy, which is contrary to the general idea of “benign” expectation when it comes to a case experiencing viral‐induced pancreatitis. Thus, this case highlights the need for clinicians to consider HEV as a potential cause of acute pancreatitis, especially in endemic regions or when conventional risk factors are not seen [5, 7, 9].

The management for pancreatitis is mainly supportive, which includes fluid resuscitation, pain control, and careful monitoring of vitals [3, 5]. Our patient was shifted to the intensive care unit due to concomitant acute liver failure and pancreatitis. There, he demonstrated gradual clinical improvement, and his recovery was uneventful; he showed improvement in liver function, and his pancreatic inflammation subsided within one month. This outcome aligns with previous reports indicating that HEV‐induced pancreatitis generally has a good prognosis in immunocompetent adults, although severe or fulminant cases may occur [5, 9].

The significance of this case lies in its diagnostic and clinical implications. First, it highlights the importance of suspecting HEV in the differential diagnosis of acute pancreatitis, particularly in patients residing in endemic regions. Early recognition of viral etiology can prevent unnecessary invasive investigations and guide appropriate supportive therapy. Also, it highlights the need for monitoring of pancreatic involvement in patients with acute viral hepatitis, as early detection of pancreatitis can change the level of care and further management. Finally, reporting such rare cases contributes to the growing body of evidence guiding clinical decision‐making in regions where HEV is endemic.

## 4 Conclusion

In conclusion, HEV should be suspected as a potential cause of acute pancreatitis. Clinicians should maintain a high index of suspicion in patients presenting with concurrent hepatic and pancreatic dysfunction, particularly in the endemic regions. Early identification and supportive management can lead to favorable outcomes, as demonstrated in our patient.

## Author Contributions

**Syed Abdur Rehman Shah:** conceptualization, data curation, methodology, writing – original draft. **Intikhab Alam:** conceptualization, project administration, writing – original draft. **Syed Noman Ali Shah:** conceptualization, data curation, supervision, writing – original draft. **Umaima Cheema:** investigation, validation, writing – original draft. **Aymar Akilimali:** conceptualization, data curation, writing – review and editing.

## Funding

The authors have nothing to report.

## Ethics Statement

Ethical approval was obtained, and the patient signed a consent form.

## Consent

Written informed consent was obtained from the patient for publication of this case report in accordance with journal guidelines.

## Conflicts of Interest

The authors declare no conflicts of interest.

## Data Availability

The data that support the findings of this study are available from the corresponding author upon reasonable request.
